# A Compact Dual-Band Frequency Selective Surface for Concurrent 5G and 6G Applications

**DOI:** 10.3390/s26165269

**Published:** 2026-08-20

**Authors:** Iftikhar ud Din, Daud Khan, Fahad Ahmed, Khaled Albaden, Tayeb A. Denidni

**Affiliations:** National Institute for Scientific Research (INRS), University du Québec, Montreal, QC H5A 1K6, Canada; daud.khan@inrs.ca (D.K.); ahmed.fahad@inrs.ca (F.A.); khaled.albaden@inrs.ca (K.A.); tayeb.denidni@inrs.ca (T.A.D.)

**Keywords:** frequency selective surfaces, dual-band, transmission, wireless communication systems

## Abstract

In this article, a dual-band frequency-selective surface (FSS) is proposed for wireless communication systems operating in the sub-6 GHz 5G band and emerging upper-mid-band frequencies considered for future wireless applications. The proposed FSS exhibits two passbands centered at 4.8 GHz and 6.9 GHz, with corresponding reflection coefficients of −33 dB and −25 dB, respectively. The associated −10 dB impedance bandwidths extend from 4.6–5.0 GHz and 6.6–7.2 GHz, providing efficient transmission within the desired operating bands. Between these passbands, the proposed structure exhibits a band-stop response centered at 5.5 GHz, where the transmission coefficient ($S_{21}$) reaches a minimum value of −40 dB over the frequency range of 5.2–5.8 GHz. This intermediate stopband suppresses unwanted signal transmission, particularly within the Wi-Fi band, thereby improving spectral selectivity and reducing potential interference in multi-band wireless communication environments. Furthermore, the proposed single-layer FSS maintains stable transmission and reflection characteristics under different polarization states and oblique incidence angles up to 60°. The measured results show good agreement with the simulated responses, demonstrating the suitability of the proposed design for applications requiring dual-band transmission and intermediate band-stop filtering in next-generation wireless communication systems.

## 1. Introduction

Frequency-selective surfaces are periodic electromagnetic structures that act as spatial filters, controlling the transmission and reflection of incident waves in free space. Their behavior is determined not only by frequency but also by the angle of incidence and the polarization of the incoming electromagnetic wave, making them inherently multidimensional. Like conventional microwave filters, FSSs can be designed to exhibit low-pass, high-pass, band-pass, or band-stop characteristics; however, band-pass and band-stop responses are more widely employed due to their effectiveness in selective transmission and interference mitigation. Owing to these properties, FSSs have found extensive applications in areas such as electromagnetic shielding, spatial filtering, radome design, wireless security, and satellite communication systems [1,2,3].

**Figure 1:**
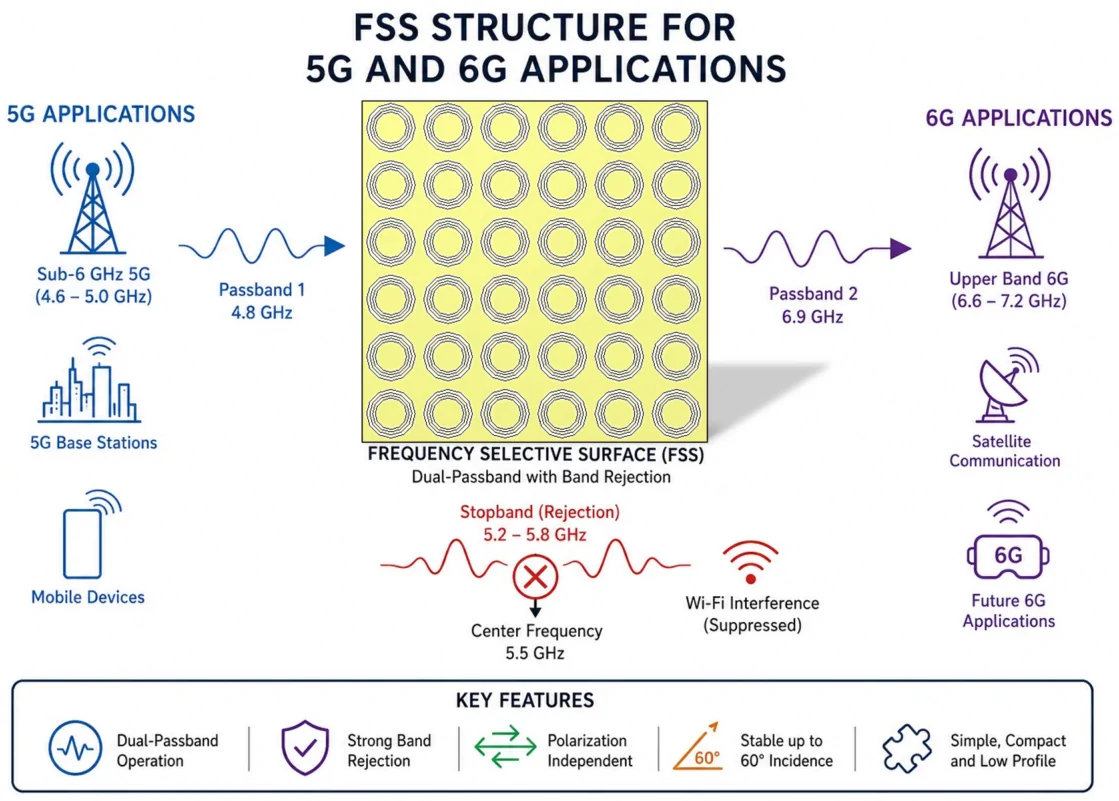
Application scenario of the proposed dual-passband FSS, enabling 5G (4.8 GHz) and 6G (6.9 GHz) transmissions while suppressing Wi-Fi interference through a stopband centered at 5.5 GHz.

In modern systems, multiple electromagnetic and optical sensors operate across a broad spectrum, spanning microwave, millimeter-wave, sub-millimeter, infrared, and optical frequency ranges. This wide spectral operation necessitates advanced filtering mechanisms to ensure system compatibility and reduce mutual interference. Consequently, there is an increasing demand for FSS designs capable of supporting multiple independent transmission bands. Such multi-band FSS structures enable efficient spectrum utilization and play a crucial role in enhancing the performance of multifunctional electromagnetic systems in both civilian and defense applications [4,5]. Traditional EMI mitigation techniques, such as metallic enclosures, wire meshes, absorbers, and shielding fabrics, are generally bulky, non-selective, and often limited to narrowband operation, which makes them unsuitable for multi-band communication systems. In contrast, FSS technology offers a compact and frequency-selective solution by using periodic structures to control electromagnetic wave propagation. FSSs can be designed to transmit desired bands while suppressing unwanted frequencies, enabling efficient interference mitigation without compromising multi-band system performance [6]. Unlike previously reported dual-band FSSs, which are generally designed for different operating frequency bands, the proposed FSS is specifically developed to provide dual-passband operation for the sub-6 GHz 5G and upper-mid-band 6G candidate frequency ranges, while simultaneously introducing an intermediate stopband for Wi-Fi suppression. Most reported FSS designs are limited by polarization sensitivity, angular instability, and insufficient rejection bandwidth, with notable degradation under TM polarization. To address these issues, this work presents a new dual-band transmissive FSS comprising two circular-slot elements on a lossy substrate. The design enables selective transmission of desired frequency bands while rejecting unwanted interference. The structure provides transmission bands at 4.8 GHz and 6.9 GHz (4.6–5.0 GHz and 6.6–7.2 GHz), suitable for sub-6 GHz 5G and emerging 6G systems [7,8]. A strong band-stop response at 5.5 GHz (5.2–5.8 GHz, $S_{21}$ −40 dB) effectively suppresses interference in the Wi-Fi band. These features make the proposed FSS well suited for multi-band systems requiring controlled transmission and interference rejection, as shown in Figure 1. The main contributions are summarized as follows: Design and implementation of a single-layer FSS unit-cell architecture to maintain a low-profile structure for 5G and emerging 6G applications. The proposed FSS unit cell exhibits polarization-independent behavior and maintains consistent transmission and reflection characteristics for incident angles up to 60°. To the best of the authors’ knowledge, this study introduces an FSS unit cell that achieves two distinct transmission bands along with a single reflection band, spanning the sub-6 GHz 5G and emerging mid-band 6G frequencies, which has not been reported in prior works. The proposed structure employs a single-layer configuration, where all resonant elements are realized on one surface without the need for multilayer stacking, resulting in a simple, cost-effective design that is straightforward to fabricate and easily integrated into wireless communication systems.

## 2. Electromagnetic Excitation Configuration and Unit-Cell Geometry of the Proposed Dual-Band FSS

**Figure 2:**
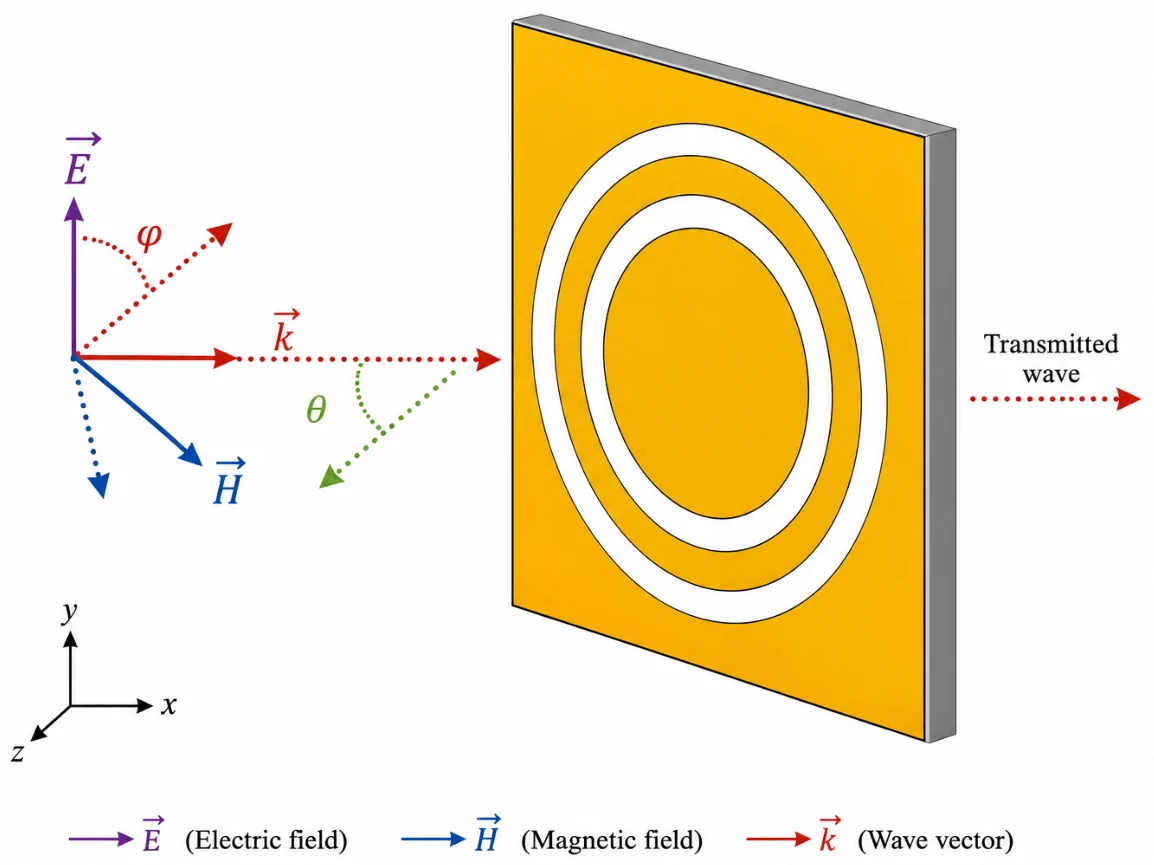
Electromagnetic excitation and unit-cell configuration of the proposed dual-band FSS, providing passbands at 4.8 GHz and 6.9 GHz and a stopband at 5.5 GHz.

Figure 2 illustrates the electromagnetic excitation configuration and operating principle of the proposed dual-band frequency-selective surface (FSS) intended for sub-6 GHz 5G and emerging upper-band 6G wireless communication systems. The incident electromagnetic wave is characterized by the electric field (*E*), magnetic field (*H*), and propagation vector (*k*), while the polarization angle (ϕ) and incidence angle (θ) define the excitation conditions used to evaluate the polarization and angular stability of the structure. The proposed FSS consists of a compact concentric resonator configuration printed on a dielectric substrate, enabling selective transmission and rejection characteristics within the desired frequency spectrum. As shown in Figure 2, the proposed structure exhibits two distinct transmission bands centered at 4.8 GHz and 6.9 GHz, corresponding to the sub-6 GHz 5G and upper-band 6G frequency ranges, respectively. The reflection coefficients reach approximately −33 dB and −25 dB at these resonances, indicating excellent impedance matching with free space. The corresponding −10 dB bandwidths extend from 4.6–5.0 GHz and 6.6–7.2 GHz, ensuring reliable operation across the targeted communication bands. The dual-passband response is achieved through the strong electromagnetic interaction among the concentric resonating elements, which support multiple resonant modes while maintaining a compact and low-profile unit-cell geometry. In addition to its dual-band transmission characteristics, the proposed FSS exhibits a strong stopband centered at 5.5 GHz. The transmission coefficient ($S_{21}$) reaches approximately −40 dB within the 5.2–5.8 GHz frequency range, resulting in the significant attenuation of undesired signals and effective suppression of WiFi-band interference. This selective rejection characteristic improves spectral isolation and enhances the coexistence of multiple wireless services operating in adjacent frequency bands. Furthermore, the rotationally symmetric geometry contributes to polarization-independent performance and stable operation under oblique incidence conditions. The proposed FSS maintains its filtering characteristics for both TE- and TM-polarized waves over a wide range of incidence angles, making it suitable for practical deployment scenarios. Owing to its dual-passband functionality, strong stopband rejection, compact structure, and robust electromagnetic performance, the proposed FSS is a promising candidate for next-generation wireless communication systems, including 5G base stations, upper-band 6G networks, satellite communication platforms, and advanced spatial filtering applications.

## 3. FSS Unit-Cell Design Simulations and Results Discussion

**Figure 3:**
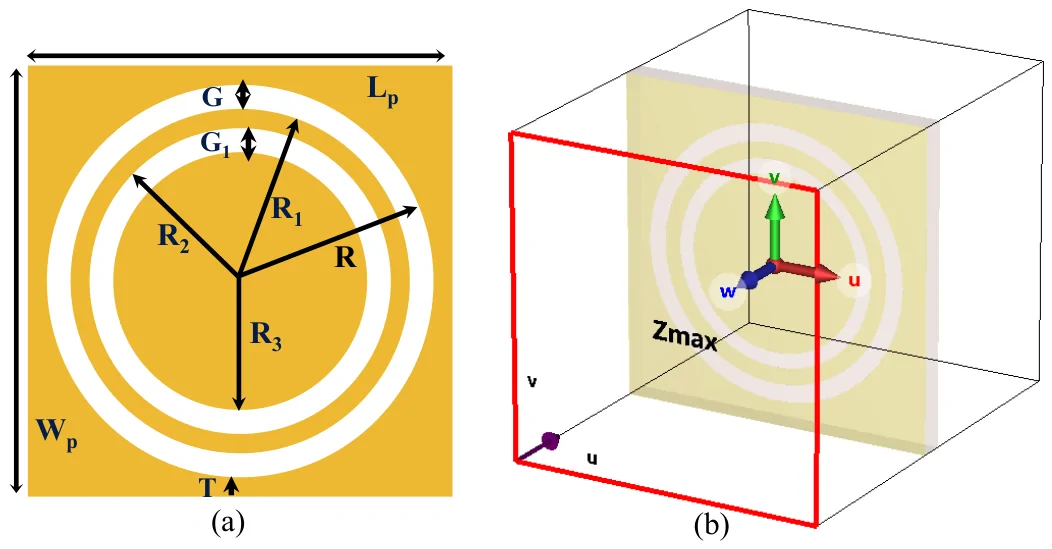
This figure illustrates the geometric configuration of the proposed metamaterial FSS unit cell: (**a**) Front View and (**b**) Full-wave simulation setup of the FSS unit cell. $R=8.5\;\mathrm{mm},\;R_{1}=7.4\;\mathrm{mm},\;R_{2}=6.4\;\mathrm{mm},\;R_{3}=5.5\;\mathrm{mm},\;H=1.52\;\mathrm{mm},\;L_{p}=W_{p}=20\;\mathrm{mm},\;W_{s}=L_{s}=20\;\mathrm{mm}.$

This section presents the design methodology of the proposed FSS unit cell. The geometrical configuration of the unit cell, including its front and perspective views, is illustrated in Figure 3a,b. The structure is realized on an RO3003 dielectric substrate with a thickness of 1.52 mm, relative permittivity $\varepsilon_{r}=3$, and loss tangent tanδ=0.0010. The FSS pattern is etched on the top surface of the substrate, while the bottom surface is kept free of metallization, resulting in a single-layer transmissive configuration. All relevant geometrical parameters are defined in Figure 3. The parameter *R* denotes the outer radius of the circular patch, while *G* represents the gap between the outer and middle resonating elements. The spacing between the middle and inner circular patches is denoted by $G_{1}$. The inner ring radius is denoted by $R_{3}$, and *T* represents the conductor width of the circular-slot square. The substrate thickness and periodicity of the unit cell are given by *H* and $W_{s}$, respectively. The proposed FSS unit cell is modeled and analyzed using CST Microwave Studio under full-wave simulation conditions. A normally incident plane wave propagating along the *z*-axis is considered, while periodic (unit-cell) boundary conditions are applied in the *x*- and *y*-directions to emulate an infinite FSS array, as shown in Figure 3b.

## 4. Design Evolution and Performance Analysis

**Figure 4:**
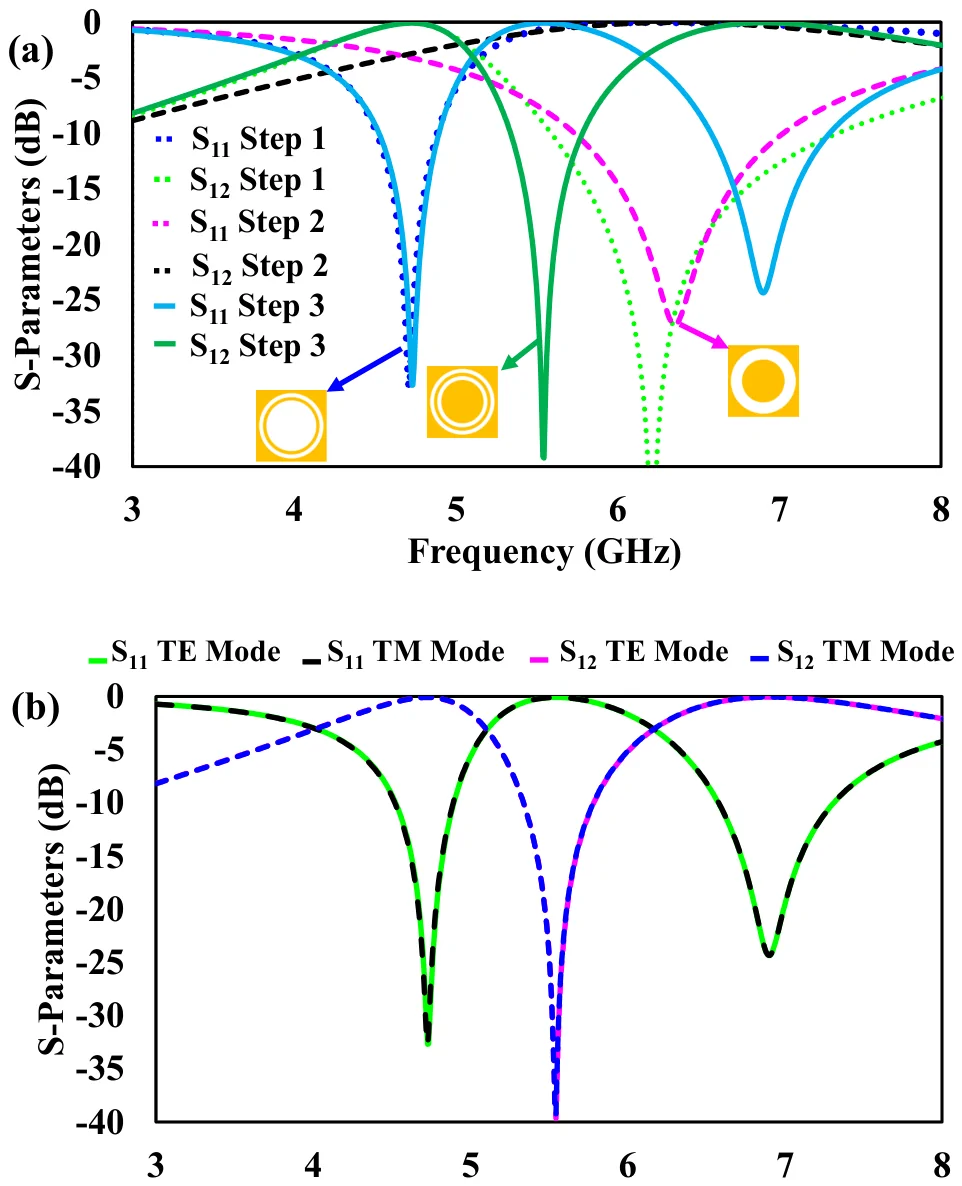
Design Evolution of the proposed unit cell: (**a**) geometrical configurations at each stage and corresponding reflection and transmission responses; (**b**) simulated reflection and transmission characteristics of the proposed structure for TE and TM modes.

The evolution of the proposed FSS unit cell is carried out in three design steps, and the corresponding reflection ($S_{11}$) and transmission ($S_{21}$) responses and the geometrical configurations are shown in Figure 4a. In Step 1, a simple circular loop is employed as the initial configuration. The $S_{11}$ response exhibits a distinct resonance within 4.6–5.0 GHz, with a deep notch below −30 dB, indicating strong impedance matching. However, the transmission coefficient ($S_{21}$) remains close to 0 dB across the band, confirming that the structure primarily supports wave transmission and behaves as a passband element without significant filtering selectivity. In Step 2, a central metallic patch is introduced within the loop, enhancing capacitive coupling and modifying the current distribution. This results in a broad transmission response spanning approximately 5.0–7.0 GHz, where $S_{21}$ approaches 0 dB, indicating a wide passband. The increased coupling leads to improved energy confinement and a more uniform transmission characteristic. In Step 3, the final geometry is obtained by integrating the outer loop (Step 1) with the inner patch (Step 2). This configuration introduces multiple coupled resonances, resulting in dual-passband behavior. Specifically, two distinct transmission bands are observed, the first in the range of 4.6–5.0 GHz and the second around 6.6–7.2 GHz, both characterized by transmission coefficients close to 0 dB. Between these bands, a stopband is formed in the range of approximately 5.2–6.0 GHz, where the $S_{11}$ response increases, indicating strong reflection. Overall, the final design exhibits multi-resonant behavior with two well-defined passbands and an intermediate stopband, providing enhanced frequency selectivity. These characteristics make the proposed FSS unit cell suitable for 5G and emerging upper-6 G frequency-selective applications.

## 5. Working Mechanism

The dual-band response of the proposed FSS originates from the interaction between two electromagnetically coupled resonators having different electrical lengths. The outer circular resonator provides the longest current path and therefore exhibits a larger equivalent inductance. Together with the distributed capacitance introduced by the adjacent metallic regions and the dielectric substrate, it forms the first LC resonator responsible for the lower-frequency transmission band centered at 4.8 GHz. In contrast, the inner circular resonator possesses a shorter electrical path, resulting in a smaller equivalent inductance and consequently generating the higher-frequency transmission band at 6.9 GHz. The narrow gaps separating the concentric resonators behave as distributed capacitive elements that store electric energy and establish strong electromagnetic coupling between the two resonators. This coupling modifies the effective impedance of the unit cell and determines the frequency separation between the two transmission bands. As the operating frequency approaches 5.5 GHz, the currents induced on the outer and inner resonators become nearly equal in magnitude but opposite in phase. The resulting destructive interference suppresses the transmitted field while enhancing the reflected field, thereby producing the observed transmission null.

## 6. Parametric Analysis of the Central Patch Radius (R3)

**Figure 5:**
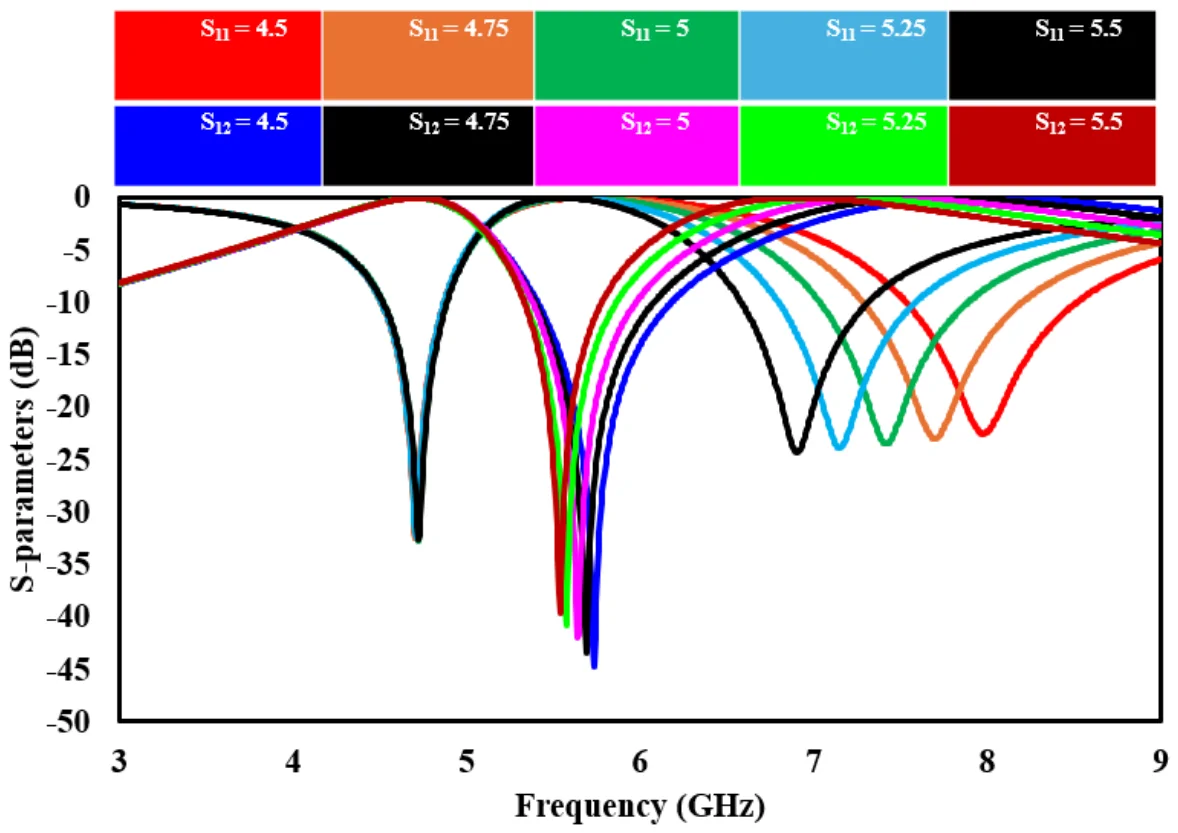
Simulated $S_{11}$ and $S_{21}$ responses for different values of the central patch radius ($R_{3}$), illustrating the independent tuning of the higher-frequency passband with minimal influence on the lower-frequency passband.

Figure 5 presents the parametric analysis of the central patch radius, $R_{3}$, to investigate its influence on the frequency-selective characteristics of the proposed FSS. During this study, $R_{3}$ was varied from 4.5 mm to 5.5 mm, while all remaining geometrical parameters were maintained at their optimized values. As observed, variations in $R_{3}$ have a negligible impact on the lower passband centered at approximately 4.8 GHz, indicating that the outer resonator predominantly determines this resonance. In contrast, the higher-frequency passband shifts significantly toward lower frequencies as $R_{3}$ increases. This behavior is attributed to the increase in the effective electrical length and equivalent capacitance of the central resonator, which lowers its resonant frequency in accordance with the LC resonance mechanism. Consequently, increasing $R_{3}$ from 4.5 mm to 5.5 mm shifts the second passband from approximately 8.0 GHz to 6.9 GHz, while the corresponding transmission response ($S_{21}$) follows the same trend. It is also evident that the intermediate stopband remains well preserved throughout the parameter variation, demonstrating that the central patch radius primarily governs the upper resonant mode with minimal influence on the lower passband. This independent tuning characteristic provides considerable design flexibility by enabling precise adjustment of the higher operating frequency without degrading the lower-band performance. Based on this analysis, an optimized value of $R_{3}=5.5$ mm was selected to achieve the desired dual-band response.

## 7. Simulated S-Parameter Performance for TE and TM Incidence

The simulated reflection ($S_{11}$) and transmission ($S_{21}$) characteristics of the proposed FSS unit cell under both TE and TM polarizations are presented in Figure 4b. The structure demonstrates a dual-bandpass response with resonances centered at approximately 4.7 GHz and 6.9 GHz, covering relevant 5G and emerging upper 6G frequency bands. The corresponding bandwidths are approximately 0.45 GHz and 0.7 GHz, respectively. In addition to the passbands, a distinct stopband is observed around 5.5 GHz, where transmission is suppressed and reflection is dominant. The transmission coefficient ($S_{21}$) remains close to 0 dB within the passbands, with an insertion loss of less than 0.2 dB, indicating efficient low-loss signal propagation. Furthermore, the ratio between the two resonance frequencies (4.7 GHz and 6.9 GHz) is approximately 1.4. This separation ensures sufficient isolation between the operating bands, allowing independent functionality across different frequency ranges. At the same time, the moderate spacing between these resonances supports compact multi-band operation, making the design suitable for integrated and frequency-agile systems.

## 8. Polarization Insensitivity Analysis

**Figure 6:**
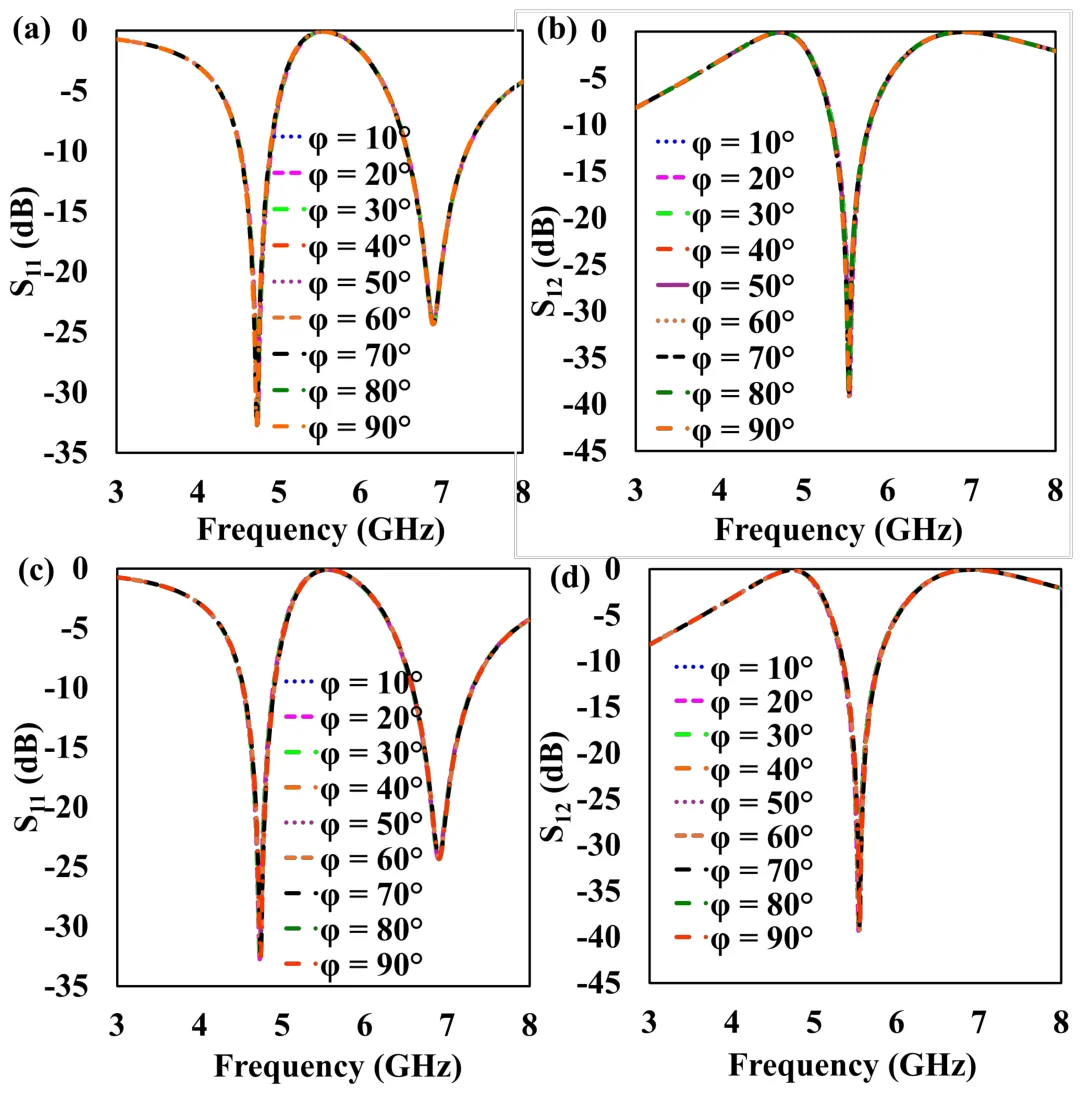
Simulated *S*-parameter variation with ϕ in TE mode and TM mode: (**a**,**c**) $S_{11}$ and (**b**,**d**) $S_{12}$.

The Polarization-independent behavior is a key requirement in FSS designs, as it ensures reliable performance in practical environments where the incident wave polarization may vary. The proposed structure attains this property due to its rotationally symmetric geometry, which preserves the resonance response for both TE and TM modes regardless of the polarization angle. This characteristic is verified in Figure 6a,c, where the S-parameters ($S_{11}$) are evaluated for polarization angles ϕ = 0°, 20°, 30°, 40°, 50°, 60°, 70°, 80° and 90° under TE and TM incidence. Similarly, Figure 6b,d present the corresponding S-parameters ($S_{21}$) for the same polarization angles in the TE and TM modes. The negligible variation in these responses confirms that the proposed design maintains stable electromagnetic performance irrespective of polarization changes.

## 9. Angular Stability

**Figure 7:**
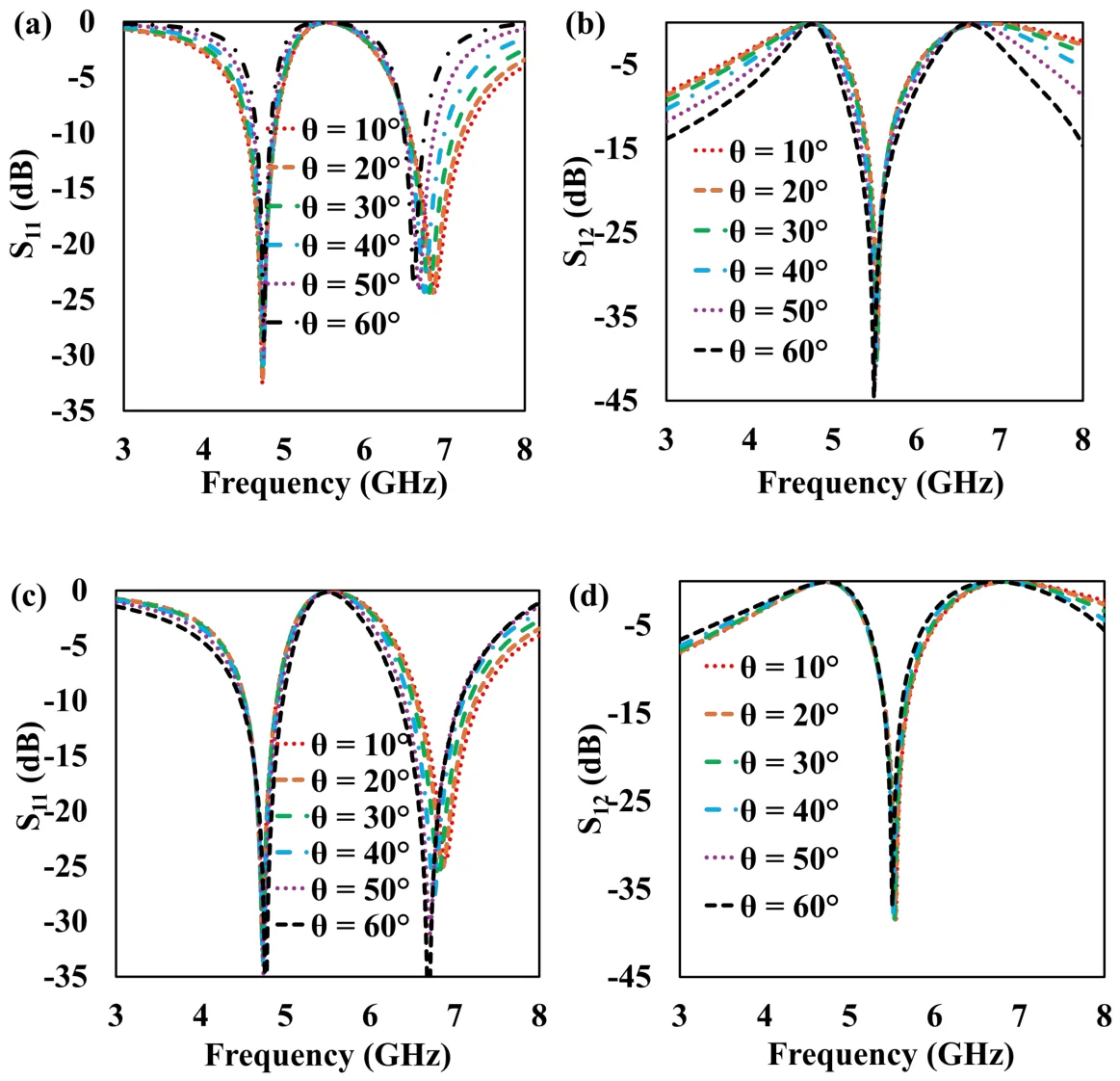
Simulated *S*-parameter variation with θ in TE mode and TM mode: (**a**,**c**) $S_{11}$ and (**b**,**d**) $S_{12}$.

The angular stability of the proposed dual-band FSS was investigated under oblique incidence for both TE and TM polarizations by varying the incidence angle from 0° to 60° in steps of 10°, as illustrated in Figure 7. Figure 7a,c present the simulated $S_{11}$ responses, while Figure 7b,d show the corresponding $S_{21}$ characteristics for both polarization modes. To provide a quantitative assessment of the angular performance, the resonant frequencies, insertion losses, and corresponding −10 dB bandwidths at each incidence angle are summarized in Table 1. The results indicate that the lower-frequency passband exhibits excellent angular stability, with only negligible resonance shift and minimal insertion-loss variation throughout the investigated angular range. In contrast, the upper-frequency passband exhibits a gradual shift in resonance accompanied by a moderate reduction in bandwidth as the incidence angle increases, particularly beyond 40°. These variations are primarily attributed to the increased effective propagation path length and changes in electromagnetic coupling between the concentric resonators under oblique incidence [9]. Nevertheless, the maximum frequency shift, insertion-loss variation, and bandwidth degradation remain limited for both TE and TM polarizations, confirming that the proposed FSS preserves its dual-band filtering characteristics, polarization-independent behavior, and satisfactory impedance matching up to an incidence angle of 60°. These results demonstrate the suitability of the proposed structure for practical free-space wireless communication applications.

## 10. Surface Currents Distribution

**Figure 8:**
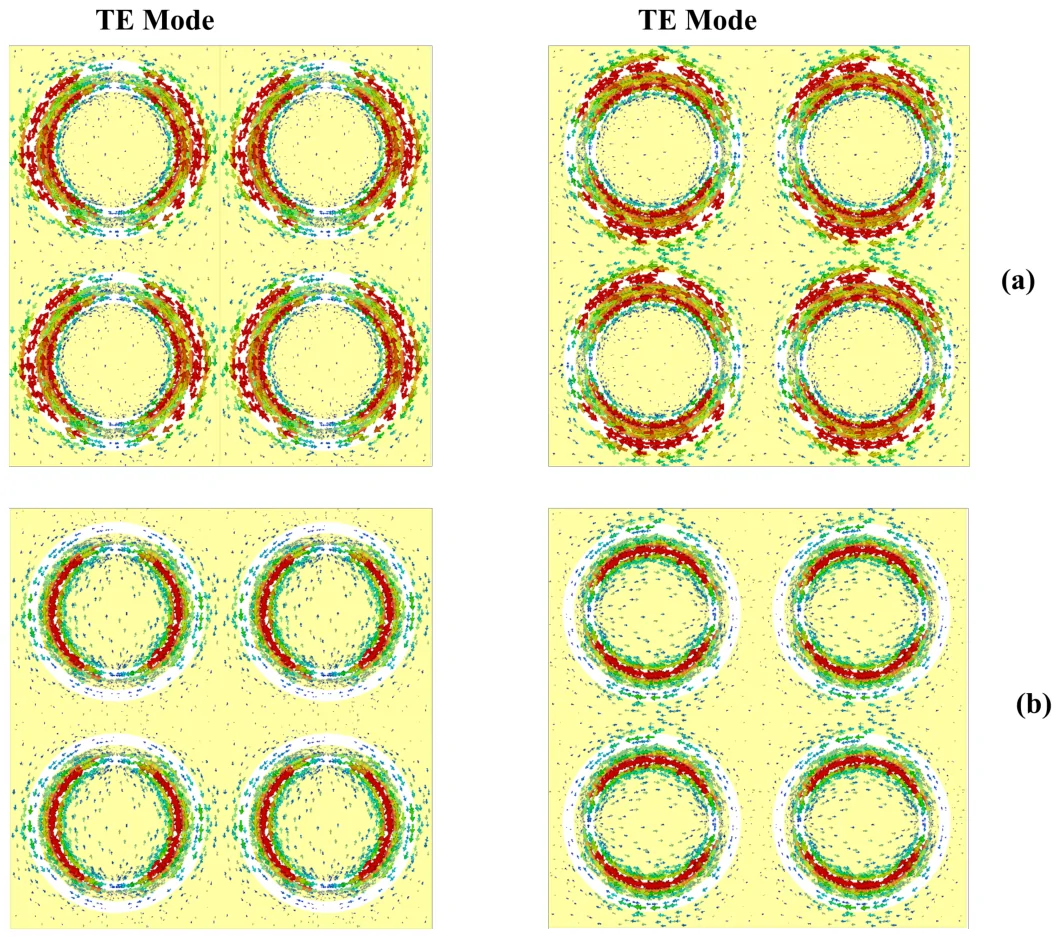
Simulated surface current distributions of the proposed bandpass FSS filter at (**a**) 4.7 GHz and (**b**) 6.9 GHz, considering both TE and TM modes.

A detailed investigation of the surface current distribution of the proposed unit cell was carried out to gain deeper insight into its resonance behavior. Figure 8 illustrates the simulated surface current distributions at 4.7 GHz and 6.9 GHz under both TE and TM modes. At 4.7 GHz, strong current concentrations are observed along the outer metallic rings, indicating that the fundamental resonance is primarily governed by the outer resonator structure. In contrast, at 6.9 GHz, the surface currents are more prominently distributed over the inner ring sections, highlighting their dominant role in generating the higher-frequency resonance. Furthermore, the current distributions remain consistent for both TE and TM polarizations, confirming the polarization-insensitive nature of the proposed FSS. These observations validate that the distinct resonant responses are controlled by different parts of the unit cell geometry.

## 11. Equivalent Circuit Analysis of the Proposed Dual-Band Pass FSS

**Figure 9:**
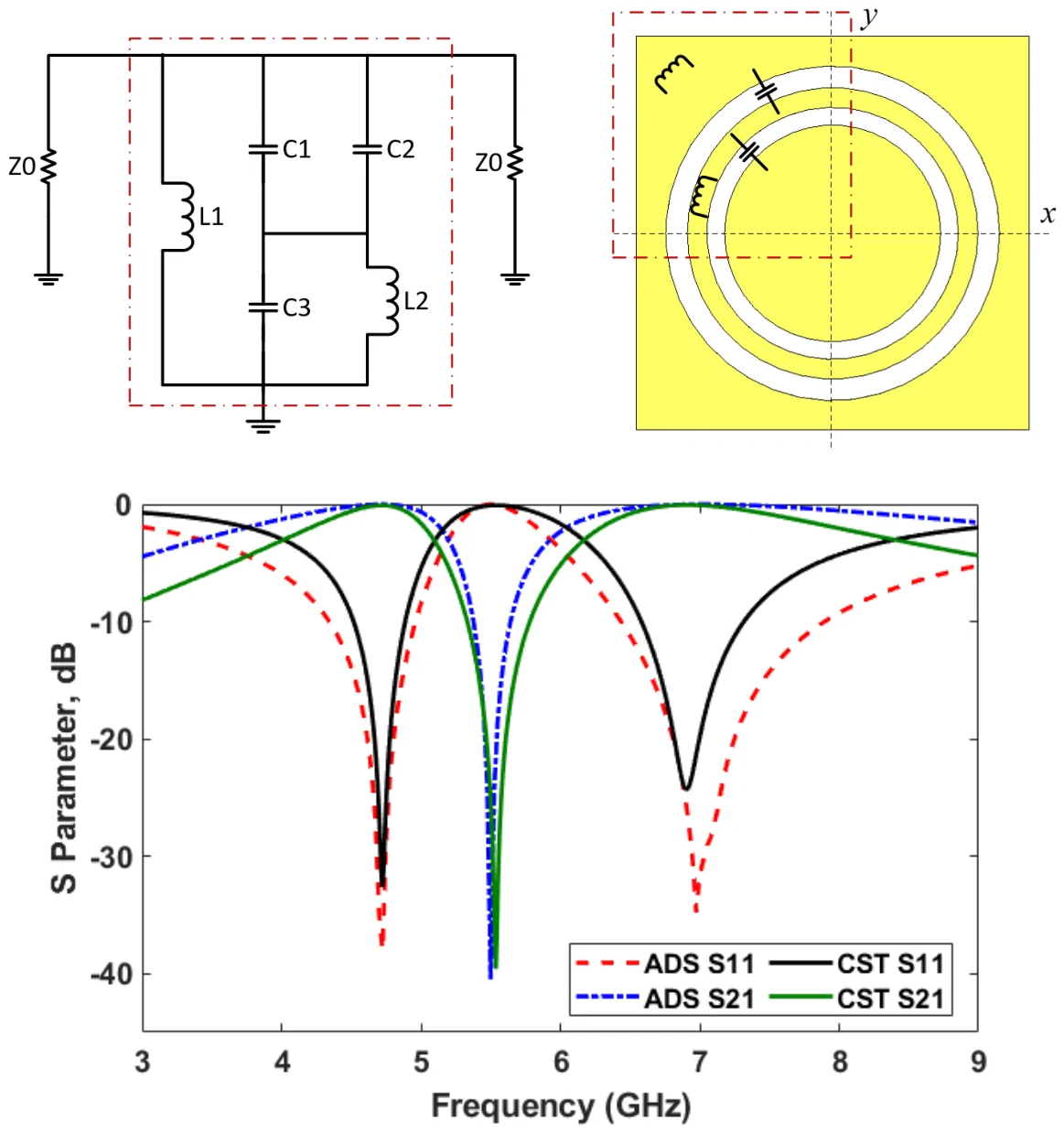
Equivalent circuit model (ECM) of the proposed FSS, the corresponding unit-cell geometry, and comparison of the ADS and CST simulated $S_{11}$ and $S_{21}$ responses, demonstrating the validity of the proposed equivalent circuit model.

The electromagnetic behavior of the proposed dual-band frequency selective surface (FSS) can be explained using the lumped-element equivalent circuit shown in Figure 9. The equivalent model consists of two inductive elements, $L_{1}$ and $L_{2}$, and three capacitive elements, $C_{1}$, $C_{2}$, and $C_{3}$, placed between two transmission regions modeled by the free-space impedance $Z_{0}$. Physically, $L_{1}$ and $L_{2}$ represent the inductive current paths associated with the outer and inner ring resonators, respectively, whereas $C_{1}$ and $C_{2}$ correspond to the capacitances introduced by the splits in the metallic rings. In addition, $C_{3}$ models the capacitive coupling between the two concentric resonators. Owing to the coupling between the two resonators, the unit cell supports two distinct transmission resonances, thereby producing a dual-band passband response. The total surface admittance of the proposed FSS is expressed as

(1) $$Y_{\mathrm{FSS}}=Y_{1}+Y_{2}$$

where $Y_{1}$ is the admittance of the first branch and $Y_{2}$ is the admittance of the second branch. The first branch is composed of the shunt inductor $L_{1}$, and its admittance is given by

(2) $$Y_{1}=\frac{1}{j\omega L_{1}}$$

where ω=2πf is the angular frequency, *f* is the operating frequency, and $j=\sqrt{-1}$. Since $C_{1}$ and $C_{2}$ are connected between the same two nodes, their equivalent capacitance is

(3) $$C_{12}=C_{1}+C_{2}$$

where $C_{12}$ is the equivalent capacitance of the split gaps. The impedance of the second branch is then written as

(4) $$Z_{2}=\frac{1}{j\omega C_{12}}+\left(j\omega L_{2}\|\frac{1}{j\omega C_{3}}\right)$$

where $L_{2}$ denotes the inductance associated with the inner resonant path and $C_{3}$ is the coupling capacitance between the two rings. The parallel combination in (4) can be simplified as

(5) $$\left(j\omega L_{2}\|\frac{1}{j\omega C_{3}}\right)=\frac{j\omega L_{2}}{1-\omega^{2}L_{2}C_{3}}.$$

Substituting (5) into (4) yields

(6) $$Z_{2}=\frac{1}{j\omega (C_{1}+C_{2})}+\frac{j\omega L_{2}}{1-\omega^{2}L_{2}C_{3}}.$$

Accordingly, the admittance of the second branch becomes

(7) $$Y_{2}=\frac{1}{Z_{2}}.$$

The transmission resonances of the FSS occur when the input impedance of the equivalent circuit approaches the free-space impedance $Z_{0}$, resulting in a minimum in $S_{11}$ and a corresponding maximum in $S_{21}$. The lower resonant frequency is primarily associated with the outer split-ring resonator because its longer current path produces a larger equivalent inductance, whereas the higher resonant frequency is governed by the inner split-ring resonator and the inter-ring coupling capacitance $C_{3}$. Substituting (2) and (7) into (1) and simplifying the resulting expression yields the total surface admittance, from which the corresponding surface impedance can be determined ($Z_{\mathrm{FSS}}=\frac{1}{Y_{\mathrm{FSS}}}$). The resonant frequencies $f_{r1}$ and $f_{r2}$ can then be obtained by solving the resulting characteristic equation.

From the simulated and circuit-based *S*-parameter responses shown in Figure 9, the two resonant frequencies are extracted from the minima of $S_{11}$ and the corresponding maxima of $S_{21}$. The first resonance is observed at approximately 4.75 GHz from CST and 4.78 GHz from ADS, while the second resonance appears at approximately 6.95 GHz from CST and 7.00 GHz from ADS. Therefore, the average resonant frequencies of the proposed dual-band FSS are approximately 4.76 GHz and 6.98 GHz. The corresponding relative deviations between the ADS equivalent circuit model and the CST full-wave simulation are only 0.63% and 0.72% for the first and second resonances, respectively. The values of $L_{1}$, $L_{2}$, $C_{1}$, $C_{2}$, and $C_{3}$, listed in Table 2, were obtained through an iterative curve-fitting process in Keysight ADS, where the lumped-element values were optimized until the equivalent circuit response closely matched the full-wave CST simulation. The good agreement between the ADS circuit model and the full-wave CST results confirms that the proposed equivalent circuit accurately captures the dual-band resonant behavior of the concentric split-ring FSS unit cell.

The reflection coefficient ($S_{11}$) exhibits two distinct resonant modes, with the ADS results (red dashed line) closely matching the CST response (black solid line), although a slight frequency shift and deeper minima are observed in ADS. The transmission coefficient ($S_{21}$) also follows a consistent trend between both tools, with a pronounced dip around the central frequency, indicating strong resonance and energy coupling. Minor discrepancies between the curves can be attributed to the simplifications inherent in the equivalent circuit model compared to the full-wave CST simulation. Overall, the results validate the accuracy of the proposed circuit model in capturing the FSS’s resonant behavior.

## 12. Novelty and Scientific Contributions

The novelty of the proposed frequency-selective surface (FSS) lies in the realization of a single-layer, fabrication-friendly transmissive structure that simultaneously provides two transmission bands at 4.8 GHz and 6.9 GHz together with a deliberately engineered intermediate rejection band centered at 5.5 GHz. Unlike conventional dual-band FSS designs that primarily emphasize multiple passbands, the proposed configuration integrates selective signal transmission and interference suppression within the same periodic surface, enabling concurrent support for sub-6 GHz 5G and upper-band 6G communications while effectively mitigating Wi-Fi interference. In addition, the rotationally symmetric geometry ensures polarization-independent operation and stable performance for both transverse electric (TE) and transverse magnetic (TM) polarizations for incidence angles up to 60°. Furthermore, the operating mechanism of the proposed FSS is comprehensively validated through full-wave electromagnetic simulations, equivalent circuit modeling, and experimental measurements, demonstrating excellent agreement between theoretical and measured results.

## 13. Measured Results

**Figure 10:**
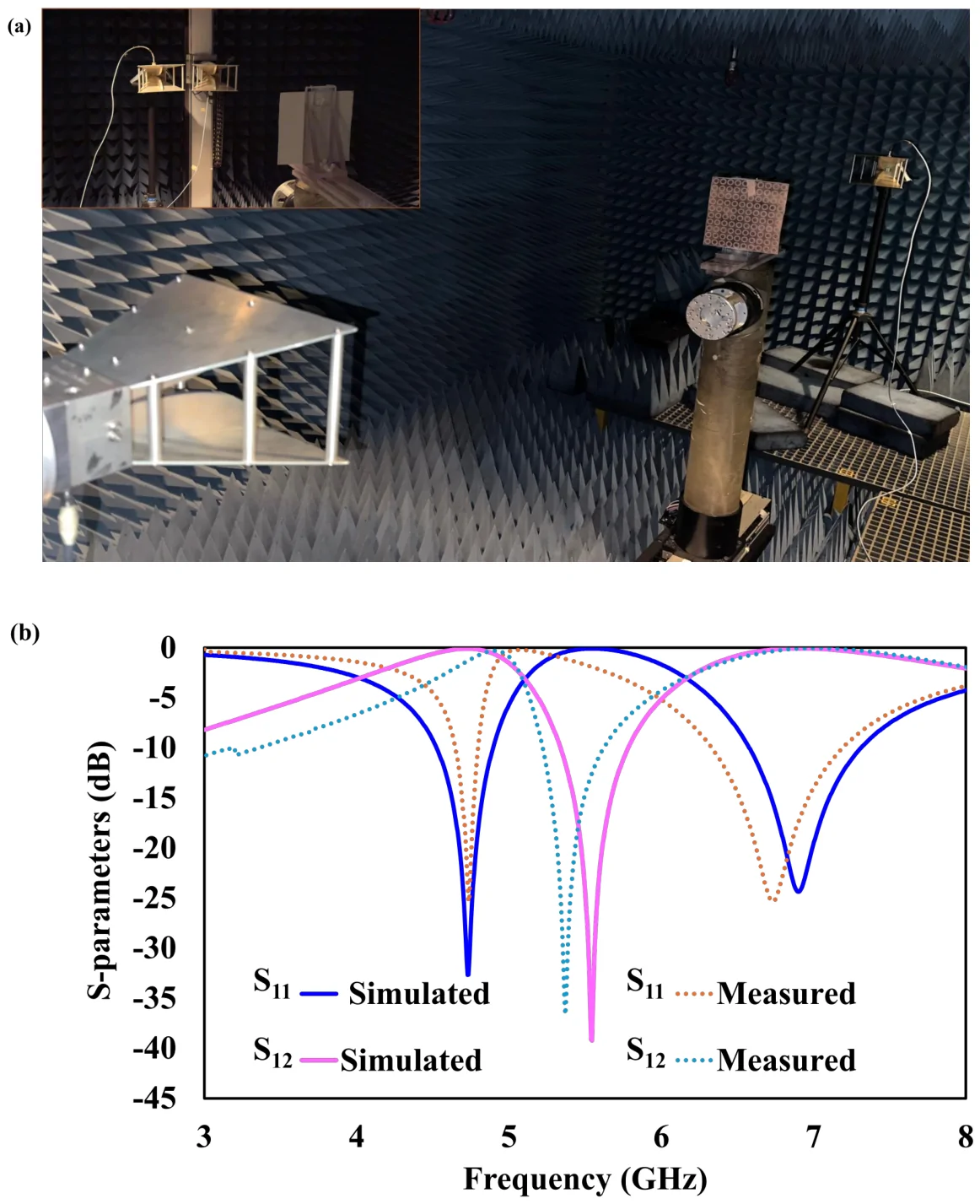
Experimental Validation of the proposed dual-band FSS. (**a**) Free-space measurement setup in the anechoic chamber using broadband horn antennas and a vector network analyzer (VNA). (**b**) Comparison between the simulated and measured reflection ($S_{11}$) and transmission ($S_{21}$) responses of the proposed FSS.

The fabricated 10 × 10 dual-band FSS prototype, with an overall size of $200\times 200\;{\mathrm{mm}}^{2}$, was experimentally characterized inside an anechoic chamber using a horn antenna-based free-space measurement setup, as illustrated in Figure 10a. The characterization began with the measurement of the reflection response. After calibrating the system, a standard gain horn antenna connected to an Agilent 8722ES vector network analyzer (VNA) was employed to transmit and receive the signal. The horn antenna was positioned approximately 2 m from the FSS prototype to satisfy the far-field condition and ensure uniform plane-wave illumination over the entire array [10]. The FSS array was placed normal to the antenna aperture, and the reflected signal ($S_{11}$) was recorded to evaluate its reflective characteristics. Subsequently, the transmission performance was investigated using two standard gain horn antennas connected to the same VNA. A reference measurement of the transmission coefficient ($S_{21}$) was first performed without the FSS sample to eliminate the effects of free-space propagation and system losses. The FSS array was then positioned midway between the transmitting and receiving antennas, and the measured transmission response was normalized with respect to the reference measurement. No time-gating technique was employed during the measurements, as the experiments were conducted inside an anechoic chamber with sufficient absorber coverage to minimize unwanted reflections. As shown in Figure 10b, the measured $S_{21}$ results closely agree with the simulated results, confirming the validity of the proposed design. The measurements verify dual-band transmission over the frequency ranges of 4.6–5.0 GHz and 6.6–7.2 GHz, together with a distinct stop band (reflective response) from 5.2 to 6.0 GHz. The overall measurement uncertainty is mainly associated with fabrication tolerances, slight dimensional variations in the etched elements, finite-array edge effects, antenna alignment errors, connector and cable losses, and the limited dynamic range of the measurement system. Minor discrepancies between the simulated and measured $S_{11}$/$S_{21}$ responses, particularly near the band edges, are primarily attributed to these factors, as well as the finite dimensions of the fabricated array compared with the infinite periodic boundary conditions assumed in the simulations and small variations in the dielectric properties of the fabricated substrate.

## 14. Comparison with State-of-the-Art Frequency-Selective Surfaces

Table 3 presents a performance comparison between the proposed frequency-selective surface (FSS) and previously reported designs. The proposed structure provides dual-band operation at 4.8 and 6.9 GHz with fractional bandwidths of 8.3% and 8.7%, respectively, while maintaining a compact unit-cell size of $0.75\times 0.75\lambda_{0}$. Furthermore, the FSS exhibits a low insertion loss of 0.2 dB and stable performance up to an incidence angle of 60°. Compared with existing designs, the proposed FSS offers a balanced combination of bandwidth, angular stability, compactness, and fabrication simplicity, making it a promising candidate for 5G and emerging 6G wireless communication applications.

## 15. Conclusions

In this article, the design and fabrication of an FSS filter with dual-bandpass and band-stop characteristics are presented. Two resonances occur at 4.8 GHz and 6.9 GHz, with reflection levels of −33 dB and −25 dB. The corresponding −10 dB bandwidths are 4.6–5.0 GHz and 6.6–7.2 GHz, covering sub-6 GHz 5G and emerging mid-band 6G applications. A sharp transmission null has been achieved at 5.5 GHz, reaching −40 dB over 5.2–5.8 GHz, enabling effective interference suppression and improved isolation, particularly in the WiFi band. The design maintains stable performance under both TE and TM polarizations up to 60° incidence. Measured results have shown good agreement with simulations, validating the approach. The filter is suitable for broadside applications such as windows and structural surfaces. 

## Figures and Tables

**Table 1:** Quantitative angular stability analysis of the proposed FSS. The IL and BW values correspond to the lower/upper passbands, respectively.

Mode	θ (°)	$f_{L}$ (GHz)	$f_{H}$ (GHz)	IL*_L_*/IL*_H_* (dB)	BW*_L_*/BW*_H_* (GHz)
TE	0	4.75	6.65	0.10/0.10	0.46/0.82
TE	10	4.75	6.67	0.10/0.12	0.46/0.80
TE	20	4.75	6.70	0.11/0.15	0.46/0.76
TE	30	4.76	6.73	0.12/0.18	0.45/0.70
TE	40	4.76	6.77	0.15/0.22	0.44/0.63
TE	50	4.77	6.82	0.18/0.30	0.43/0.56
TE	60	4.78	6.88	0.22/0.45	0.42/0.49
TM	0	4.75	6.67	0.10/0.10	0.47/0.82
TM	10	4.75	6.69	0.10/0.12	0.47/0.80
TM	20	4.75	6.71	0.11/0.14	0.47/0.77
TM	30	4.76	6.74	0.12/0.16	0.46/0.74
TM	40	4.76	6.77	0.14/0.20	0.46/0.70
TM	50	4.77	6.80	0.17/0.25	0.45/0.66
TM	60	4.78	6.84	0.20/0.30	0.44/0.62

**Table 2:** Equivalent Circuit Parameters.

Component	$L_{1}$	$L_{2}$	$C_{1}$	$C_{2}$	$C_{3}$	$Z_{0}$
Value	0.686 nH	0.1 nH	0.1 pF	1.1 pF	7.18 pF	377 Ω

**Table 3:** Comparison of the proposed FSS with reported designs.

Ref.	Freq. (GHz)	BW (%)	UC Size ($\lambda_{0}\times \lambda_{0}$)	IL (dB)	Ang. (°)	Fab.	Metal	Layers
[11]	0.9/1.8/2.1	NA	0.15 × 0.15	–	50	PCB	No	1
[12]	3.79/8.34/12.52	52.8/13.7/19.7	0.09 × 0.09	3	50	PCB	No	1
[13]	7.7/12.8/18.9	0.12/0.12/0.07	0.13 × 0.13	0.5	60	PCB	No	3
[14]	8.1/9.8/11	NA	0.55 × 0.55	3.26/2.68/1.12	60	PCB	No	1
[15]	9.8/10.9	NA	0.39 × 0.39	NA	45	PCB	Yes	1
[16]	12/18	3.3/1.6	0.60 × 0.60	2.8/2.6	15	NA	Yes	4
[17]	10/15.2	3.5/3.2	0.57 × 0.57	1	20	EDM	Yes	1
[18]	19.95/29.75	2.5/1.7	0.63 × 0.63	0.7	NA	Water-jet	Yes	3
[19]	3.5	39.7	0.25 × 0.25	1.65	40	Water-jet	Yes	2
[20]	10.5	30.8	0.32 × 0.32	1	45	Laser	Yes	2
[21]	27.9	27.2	0.54 × 0.54	0.8	45	Chem. Etch.	Yes	3
[22]	28	14.3	0.80 × 0.80	0.6	30	Chem./Mill.	Yes	2
[23]	30	17	0.50 × 1.40/0.30	NA	20	3D Print	No	1
[24]	2.33/3.40	5.32/9.09	0.36 × 0.36	0.6	30	PCB/Mill.	No	3
[25]	8.9–19.6	62	0.29 × 0.29	0.06	45	PCB	No	3
[26]	5.15/8.33	52.45/35.98	0.171 × 0.171	0.39/0.19	80	PCB	No	2
[27]	18.3–37.2	68.1	0.17 × 0.17	<3	±40	PCB	No	3
[28]	8.7–14.8	50	0.24 × 0.24	−	±75	-	No	1
**This work**	**4.8/6.9**	**8.3/8.7**	**0.37 × 0.37**	**0.2**	**60**	**Chem. Etch.**	**No**	**1**

## Data Availability

The original contributions presented in this study are included in the article. Further inquiries can be directed to the corresponding author.
